# Diverticule de Zenker chez une patiente en hémodialyse

**DOI:** 10.11604/pamj.2020.37.121.24932

**Published:** 2020-10-05

**Authors:** Mbengue Mansour, Fall Alioune Badara, Lemrabott Tall Ahmed, Ndiaye Babacar, Faye Maria, Fall Khodia, Faye Moustapha, Ba Bakary, Diagne Seynabou, Keita Niakhaleen, Ba Mamadou Aw, Dieng Ameth, Ndongo Modou, Sy Abou, Ka El Hadji Fary, Diouf Boucar

**Affiliations:** 1 Service de Néphrologie de l´Hôpital Aristide Le Dantec, Université Cheikh Anta Diop de Dakar, Dakar, Sénégal

**Keywords:** Diverticule de Zenker, hémodialyse, transit œsogastroduodénale, dénutrition protéino-énergétique, pneumopathie, Zenker’s diverticulum, hemodialysis, esophagogastroduodenal transit, protein-energy malnutrition, pneumopathy

## Abstract

Le diverticule de Zenker est une pathologie rare et généralement bénigne. Sa présence chez le malade en hémodialyse a des implications thérapeutiques et pronostiques et constitue un facteur de risque de morbi-mortalité du fait de ses complications comme la dénutrition protéino-énergétique et la pneumopathie. Nous rapportons le cas d´un diverticule de Zenker diagnostiqué chez une patiente hémodialysée chronique. Il s´agissait d´une patiente âgée de 61 ans, qui était reçue pour une hémorragie digestive haute accompagnée d´une dysphagie. L´examen physique trouvait une altération de l´état général avec une perte de poids estimée à 5 kg en 3 mois. Le transit œsogastroduodénal objectivait un refoulement de la portion cervicale de l´œsophage par une volumineuse image d´addition hétérogène dont le pôle supérieur se situe au niveau de la jonction pharyngo-œsophagienne. Le diagnostic d´un diverticule de Zenker avait été retenu. Une diverticulectomie par voie cervicale externe avait été réalisé. La patiente est décédée dans les suites postopératoires précoces d´une pneumopathie d´inhalation. Le diverticule de Zenker est une pathologie rare qui est généralement bénigne mais sa présence chez le malade hémodialysé chronique augmente sa morbi-mortalité.

## Abstract (en)

Zenker’s diverticulum is a rare and generally benign condition. Its occurrence in a hemodialysis patient has therapeutic and prognostic implications and is a risk factor for mortality and morbidity due to its complications, such as protein-energy malnutrition and pneumonitis. We here report a case of Zenker’s diverticulum diagnosed in a chronic haemodialysis patient. The study involved a 61-year-old female patient admitted with upper gastrointestinal bleeding associated with dysphagia. Physical examination showed alteration of general condition and the patient reported an average weight loss of 5 kg in 3 months. Esophagogastroduodenal transit was characterized by dilatation of the cervical esophagus, appearing as a large heterogeneous niche whose upper pole was at the level of the pharyngoesophageal junction. The diagnosis of Zenker’s diverticulum was retained. Diverticulectomy by cervical incision was performed. The patient died due to inhalational lung disease in the early postoperative period. Zenker diverticulum is a rare, generally benign disease, but in patients undergoing chronic haemodialysis, it increases mortality and morbidity.

## Introduction

Le diverticule de Zenker est une hernie de la muqueuse pharyngée postérieure développée au niveau de la jonction pharyngo-œsophagienne, entre les fibres du muscle constricteur inférieur du pharynx et du muscle crico-pharyngée. C´est une pathologie rare, intéressant 1% de la pathologie œsophagienne [1]. Sa présence chez le malade en hémodialyse a des implications thérapeutiques et pronostiques. Le traitement endoscopique et la diverticulopexie semblent être mieux supportés sur ce terrain. L´aggravation de la dénutrition proteino-énergétique ainsi que la susceptibilité aux infections déjà existantes sur ce terrain accroissent la morbi-mortalité de cette pathologie. Nous rapportons le cas d´un diverticule de Zenker diagnostiqué chez une patiente hémodialysée chronique dans un contexte d´hémorragie digestive haute.

## Patient et observation

Mme F N, âgée de 61 ans, hémodialysée chronique depuis 5 ans pour une maladie rénale chronique stade V par néphroangiosclérose bénigne, qui avait été hospitalisée au service de néphrologie de l´hôpital Aristide Le Dantec de Dakar pour une hémorragie digestive haute. A l´admission, l´interrogatoire trouvait une dysphagie aux aliments solides et une toux sèche. L´examen physique trouvait une altération de l´état général avec une perte de poids estimée à 5 kg en 3 mois, un méléna et une tuméfaction latérocervicale droite non mobile à la déglutition. Le reste de l´examen physique était normal. Les constantes étaient pour la pression artérielle à 150/90 mmhg, le pouls à 112 battements/mn, température à 39,2°C, la fréquence respiratoire à 24 cycles/mn. A la paraclinique, l´hémogramme montrait une anémie à 7,2 g/dl, une hyperleucocytose à 11000/mm^3^, la protéine C réactive à 260 mg/l.

**Figure 1:**
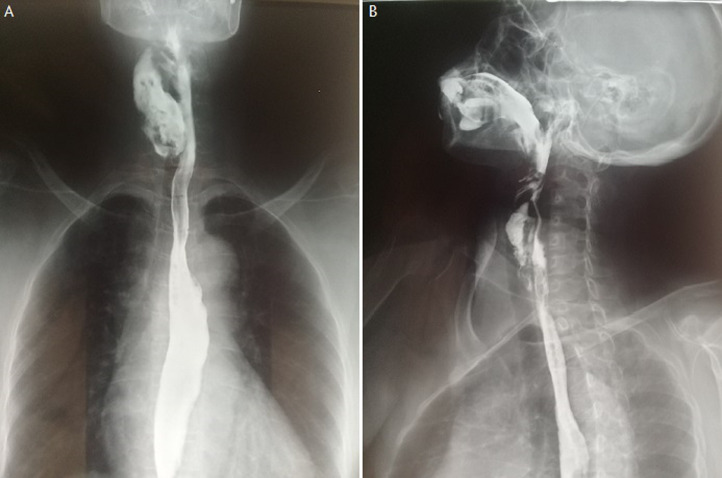
A) transit œsogastroduodénale montrant le diverticule de Zenker (vue de face); B) transit œsogastroduodénale montrant le diverticule de Zenker (vue de profil)

La calcémie corrigée était à 102,3 mg/l, la parathormone intacte était élevée à 13 fois la normale. La fibroscopie œsogastroduodénale montrait un diverticule œsophagien à 20 cm des arcades dentaires dont le fond était tapissé de lésions ulcérées et hémorragique. Le transit œsogastroduodénale objectivait un refoulement de la portion cervicale de l´œsophage par une volumineuse image d´addition hétérogène, étagé de C3 à C7 dont le pôle supérieur se situe au niveau de la jonction pharyngo-œsophagienne (Figure 1, Figure 2). La tomodensitométrie cervicale montrait un diverticule avec un collet plus ou moins étroit et un fond qui est latéro-thyroïdien droit siégeant à hauteur du pôle supérieur du lobe thyroïdien. Le diagnostic d´un diverticule de Zenker compliqué d´hémorragie et de surinfection avait été retenu. La patiente avait été mise sous etamsylate en injectable et des transfusions de concentrés globulaires ont été faites. Une diverticulectomie par voie cervicale externe avait été réalisé. La patiente est décédée dans les suites postopératoires précoces d´une pneumopathie d´inhalation.

## Discussion

Le diverticule de Zenker est un diverticule pharyngo-œsophagien situé au niveau du muscle cricopharyngien. Il a été décrit pour la première fois en 1769 par le chirurgien anglais Abraham Ludlow, après une découverte sur une autopsie. Ce diverticule a ensuite pris le nom du pathologiste Zenker, qui a publié par la suite une série de cas en 1877 [2]. Il s´agit d´un diverticule de pulsion provoqué par une hyperpression répétée du bol alimentaire au niveau de la bouche de l´œsophage. La pathogénie implique un dysfonctionnement du muscle crico-pharyngien par défaut de relâchement au moment du passage des aliments et une faiblesse de la paroi pharyngée postérieure à la jonction du muscle constricteur inférieur du pharynx et du muscle crico-pharyngien [3]. Le diverticule se remplit de résidus alimentaires, ce qui entraîne une compression œsophagienne à l´origine d´une dysphagie. Il se déclare, classiquement chez le sujet âgé de 65 à 70 ans, particulièrement de sexe masculin [4]. La symptomatologie clinique est marquée par une dysphagie de siège cervical, une halitose, des régurgitations, une toux liée à la stase alimentaire, et un risque d´inhalation [2]. Une tuméfaction basicervicale molle et fluctuante souvent gauche, se réduisant à la palpation avec un bruit hydroaérique et des régurgitations, correspondant à certains diverticules volumineux [3].

Le diagnostic est établi par un transit pharyngo-œsophagien qui met en évidence une image d´addition du tiers supérieur de l´œsophage caractéristique comme présentée par notre patiente. Quant à l´œsophagoscopie, elle n´est pas indispensable au diagnostic. Elle est indiquée en cas de doute diagnostique ou de suspicion de cancérisation. Son risque majeur est la perforation [5]. L´évolution spontanée du diverticule de Zenker se fait vers la dénutrition progressive. Et ceci est important à savoir car peut aggraver la dénutrition qui existe souvent chez le malade en hémodialyse comme c´était le cas chez notre patiente. Les mécanismes de la dénutrition protéino-énergétique en hémodialyse sont en rapport principalement à l´insuffisance d´apports nutritionnels, l´accumulation des toxines urémiques anorexigènes, l´hypercatabolisme (dialyse, infection, pathologies associées...) et l´inflammation chronique. La dénutrition protéino-énergétique est un facteur indépendant aggravant la mortalité et la morbidité dans les pathologies rénales chroniques. La dénutrition est particulièrement fréquente lors de pathologies chroniques d´organes telles que l´insuffisance rénale, avec une incidence majeure au stade de la dialyse [6]. La pneumopathie d´inhalation fait aussi la gravité du diverticule de Zenker, d´autant plus que le sujet est âgé [4]. Le risque et la gravité de cette pneumopathie sont beaucoup plus élevés chez le patient hémodialysé chronique car ce dernier a une susceptibilité aux infections, qui serait lié à l´immunodépression que crée l´insuffisance rénale, à l´environnement de l´unité d´hémodialyse propice au risque infectieux, et à l´hémodialyse elle-même [7]. Enfin, l´évolution peut être marquée par une dégénérescence maligne en carcinome épidermoïde qui peut être vue dans moins de 1% des cas. Elle survient souvent sur des diverticules évoluant depuis longtemps [8].

Les approches chirurgicales sont la diverticulectomie à ciel ouvert avec myotomie cricopharygienne, la diverticulopexie avec myotomie cricopharygienne et la diverticulectomie par voie endoscopique [9]. La diverticulectomie avec myotomie cricopharygienne est une technique très efficace et qui permet de faire l'examen pathologique de la pièce opératoire. Cependant, elle présente des inconvénients qui sont une intervention chirurgicale et une hospitalisation plus longue et une alimentation retardée. Le taux de mortalité par diverticulectomie avec myotomie cricopharygienne est plus élevé pouvant aller jusqu´à 9,5%. La morbidité, qui survient chez 4 à 47% des patients, comprend la paralysie récurrente du nerf laryngé, la sténose œsophagienne, la médiastinite, la fistule pharyngocutanée, l´hématome, la perforation œsophagienne [9]. La diverticulotomie endoscopique a comme avantages son efficacité un temps chirurgical et une hospitalisation plus courte, un retour rapide à l'alimentation orale, moins de traumatisme tissulaire. Une étude de Bonavina a montré que le taux de réussite était plus élevé chez les patients âgés de 70 ans ou plus et qui avaient tendance à avoir des diverticules plus gros [10]. Le taux de mortalité pour cette procédure est plus faible ne dépassant pas 1%. La morbidité, qui survient chez 10 à 31% des patients est plus faible. Pour la diverticulopexie avec myotomie cricopharygienne, les patients plus âgés avec de très grands diverticules de Zenker (> 6 cm) qui ne peuvent pas tolérer une diverticulectomie sont les meilleurs candidats car il n'y a pas de division de l'œsophage, du pharynx ou du diverticule, et il n'y a pas de ligne de suture [8]. En résumé, la diverticulotomie endoscopique et la diverticulopexie avec myotomie cricopharygienne semblent être selon la littérature, les procédures les mieux supportées chez les personnes âgées et ayant des comorbidités notamment les malades hémodialysés.

## Conclusion

Le diverticule de Zenker est une pathologie rare qui est généralement bénigne. Le traitement endoscopique et la diverticulopexie semblent être mieux supportés chez les patients ayant des comorbidités notamment les malades hémodialysés. L´aggravation de la dénutrition ainsi que la susceptibilité aux infections déjà existantes sur ce terrain accroissent la morbi-mortalité de cette pathologie.
